# Game-based training of flexibility and attention improves task-switch performance: near and far transfer of cognitive training in an EEG study

**DOI:** 10.1007/s00426-017-0933-z

**Published:** 2017-12-20

**Authors:** Kerwin J. F. Olfers, Guido P. H. Band

**Affiliations:** 1 Leiden Institute for Brain and Cognition (LIBC), Leiden, The Netherlands; 2 Cognitive Psychology Unit, Institute of Psychology, Leiden University, Leiden, The Netherlands

## Abstract

There is a demand for ways to enhance cognitive flexibility, as it can be a limiting factor for performance in daily life. Video game training has been linked to advantages in cognitive functioning, raising the question if training with video games can promote cognitive flexibility. In the current study, we investigated if game-based computerized cognitive training (GCCT) could enhance cognitive flexibility in a healthy young adult sample (N = 72), as measured by task-switch performance. Three GCCT schedules were contrasted, which targeted: (1) cognitive flexibility and task switching, (2) attention and working memory, or (3) an active control involving basic math games, in twenty 45-min sessions across 4–6 weeks. Performance on an alternating-runs task-switch paradigm during pretest and posttest sessions indicated greater overall reaction time improvements after both flexibility and attention training as compared to control, although not related to local switch cost. Flexibility training enhanced performance in the presence of distractor-related interference. In contrast, attention training was beneficial when low task difficulty undermined sustained selective attention. Furthermore, flexibility training improved response selection as indicated by a larger N2 amplitude after training as compared to control, and more efficient conflict monitoring as indicated by reduced Nc/CRN and larger Pe amplitude after training. These results provide tentative support for the efficacy of GCCT and suggest that an ideal training might include both task switching and attention components, with maximal task diversity both within and between training games.

## Introduction

Skilled video game players effortlessly switch between many actions, rules, objectives and targets, often faster than the untrained eye can follow. In particular, the fast-paced action video game genre (AVG), including first-person shooters, requires and fosters such impressive feats of multitasking and cognitive flexibility (Basak, Boot, Voss and Kramer, 2008; Boot, Blakely and Simons, 2008; Colzato et al., 2010). As cognitive flexibility can be a limiting factor for performance in daily life (e.g., picking up work after an interruption), and because some populations (e.g., older adults) are particularly hampered by reduced flexibility, there is demand for ways to enhance cognitive flexibility. Thus, the question is raised if active training with video games can be used to promote cognitive flexibility.

Notably, several studies have demonstrated the potential of AVGs to causally enhance performance on untrained task-switch measures, after as little as 3 weeks of training and for various age ranges (Basak et al., 2008; Colzato et al., 2010; Strobach, Liepelt, Schubert, & Kiesel, 2012; Wang et al., 2016). However, others studies have failed to find such improvements (e.g., Boot et al., 2008), and in a broader sense a meta-analysis by Powers, Brooks, Aldrich, Palladino and Alfieri (2013) showed negligible effects of gaming on executive functions in experimental studies. Whether games succeed in enhancing flexibility may depend on their composition: dedicated gamified computerized cognitive training (GCCT) can intensify exposure to task-switching exercises, serving as deliberate practice (Ericsson et al., 1993), while motivation is maintained by the engaging properties of game elements such as immediate reward and adaptive challenge (Green & Seitz, 2015; Hattie, 2009).

Unfortunately, current evidence for the efficacy of (G)CCTs is rather inconsistent. While learning effects are readily found (i.e., improvements on the trained games), improvements on untrained measures of the targeted cognitive function (near-transfer) or non-targeted cognitive functions (far-transfer) are frequently absent (Buitenweg, Murre, & Ridderinkhof 2012; Lampit, Hallock, & Valenzuela, 2014; Owen et al., 2010; Simons et al., 2016). However, positive results have also been reported (Anguera et al., 2013), including for task-switching performance (Gajewski & Falkenstein, 2012). Together, these observations establish the need to identify which aspects of GCCTs are required to effectively target cognitive flexibility, and which methods should be used to investigate these.

### Flexibility measures

Cognitive flexibility is generally assessed by task-switch (TS) paradigms (Allport, Styles, & Hsieh, 1994 ; Rogers & Monsell, 1995), requiring responses to alternating rule-sets, e.g., for digit–letter pairs indicate whether the letter is a vowel or consonant, or whether the digit is even or uneven. Switches between rule-sets elicit longer reaction times (RT) and reduced accuracy (ACC) as compared to repeats, also known as local switch costs. Switch costs are assumed to represent both proactive processes such as task-set (re)configuration (Rogers & Monsell, 1995) and reactive cognitive control processes such as suppression of interfering stimulus associations, conflict resolution and response selection (Kiesel et al., 2010; Koch, Gade, Schuch, & Philipp, 2010; Vandierendonck, Liefooghe, & Verbruggen, 2010). For example, a short rather than long response–stimulus interval (RSI) allows for less preparatory task-set (re)configuration, thus inducing greater switch costs. The interference between rule-sets of the separate tasks is also known as crosstalk (Rogers & Monsell, 1995), and maximizes costs when the distractor (e.g., the letter during a digit trial) provides conflicting response affordances. However, even performance on neutral distractor trials can suffer from carryover effects if non-neutral distractor trials are present in the same block (Karayanidis, Coltheart, Michie, & Murphy, 2003; Rogers & Monsell, 1995). Therefore, to isolate GCCT effects on switch performance without this carryover requires blocks which feature only neutral distractor trials (no-crosstalk). Another frequent measure in TS paradigms are mixing costs, which are generally defined as the performance costs of mixed-blocks containing both switch and repeat trials in comparison to pure blocks containing only repeat trials (Rogers & Monsell, 1995). Mixing costs can be attributed to factors such as interference resolution in the current trial, working memory management of multiple task-sets within the same block or even more cautious response tendencies (Los, 1996; Monsell, 2003; Philipp, Kalinich, Koch, & Schubotz, 2008). The question then is which of these costs can be reduced by GCCTs.

### Targeted training

One of the few CCT studies that specifically reported reduced local switch costs was by Karbach and Kray (2009). Perhaps tellingly, their participants trained on actual task-switch paradigms. Thus, the inclusion of task-switch paradigms in training might be crucial to target local switching costs. Alternatively, training-induced reductions in mixing costs have been found in several studies (Gajewski & Falkenstein, 2012; Kray, Karbach, Haenig, & Freitag, 2011; Minear & Shah, 2008). Notably, some of these cognitive training interventions did not specifically target cognitive flexibility or task switching. An explanation might be that these training effects are driven by increased control of selective attention (Karle, Watter, & Shedden, 2010), or enhanced working memory resources (Pereg, Shahar, & Meiran, 2013). As working memory and attentional processes presumably underlie many different cognitive functions and are considered to be intimately related (Awh, Vogel, & Oh, 2006), a GCCT focusing on these two aspects might yield transfer effects to task-switching performance. While in principle GCCTs can be designed to specifically target cognitive functions, few studies have directly compared the effects of different GCCT approaches. A notable exception is Anguera and colleagues (2013) in which multitask training was found to induce improvements in attention and working memory in comparison to a single-task training. In sum, it would be highly relevant to investigate whether a targeted switching training versus an attention and working memory GCCT induces differential improvements in task switching.

### Task-switch ERPs

If targeted GCCTs differentially impact cognitive flexibility, event-related brain potentials (ERPs) could further elucidate the neurocognitive origins in aspects such as general attentional processes, conflict resolution, response selection or conflict and error monitoring. Multiple ERP components have previously been associated with the various processing stages during task switching (Friedman, Nessler, Johnson, Ritter, & Bersick, 2007; Karayanidis et al., 2003; Poljac & Yeung, 2014). For target-locked potentials, the negative going N2 has been linked to decision processes (Jackson, Jackson, & Roberts, 1999; Ritter, Simson, Vaughan, & Macht, 1982) and response conflict (Bartholow et al. 2005; Nieuwenhuis, Yeung, van den Wildenberg, & Ridderinkhof, 2003). More specifically, N2 has been related to selecting the appropriate response to a target stimulus, as determined by the relevant task rule (Gajewski, Kleinsorge, & Falkenstein, 2010; Swainson et al., 2003). Importantly, Gajewski and Falkenstein (2012b) found enhanced N2 amplitude on a task-switch paradigm, after a 4-month cognitive training as compared to both an active and a passive control group. Additionally, a study by Friedman and colleagues (2007) revealed that young adults exhibit increased P3b during trials that require extra attention, such as switch trials or trials with incompatible distractors. This increased P3b amplitude during difficult trials was also greater for young adults as compared to older adults, who instead seemed to allocate the same amount of resources regardless of trial type. Notably, Gajewski and Falkenstein (2012b) also identified a larger P3b component following stimulus presentation for the cognitive training group, suggesting a general improvement in available cognitive resources.

For response-locked potentials, the error-related negativity (Ne/ERN) directly following incorrect responses is assumed to reflect error detection processes that may promote subsequent allocation of additional cognitive resources (Band, van Steenbergen, Ridderinkhof, Falkenstein, & Hommel, 2009; Falkenstein, Hohnsbein, Hoormann 1990). Gajewski and Falkenstein (2012) found the Ne/ERN to be enhanced after cognitive training as compared to passive and active control, and suggested this to indicate improved error detection following improvements in response selection. A response-locked negativity can also be seen after correct trials: the Nc/CRN (Ford, 1999; Vidal, Burle, Bonnet, Grapperon, & Hasbroucq, 2003) is most pronounced during high-conflict trials (e.g., switch trials or incompatible distractor trials), and is thought to represent response and conflict monitoring processes (Allain, Carbonnell, Falkenstein & Vidal, 2004; Bartholow et al., 2005), and the ability to develop adaptive response strategies (Eppinger, Kray, Mecklinger, & John, 2007). In contrast to the Ne/ERN, a higher Nc/CRN amplitude seems to reflect suboptimal monitoring or stimulus–response mapping. For instance, while younger adults show Nc/CRN primarily on incompatible trials during task switching, older adults show Nc/CRN independent of trial type (Eppinger et al., 2007). Moreover, enhanced Nc/CRN components have been found in patients with frontal lobe lesions, suggesting an impairment in the appropriate stimulus–response mapping (Gehring & Knight, 2000). In addition to negative going components, a late positive deflection following error responses (Pe) has been related to error evaluation processes, such as updating of response strategies and increased allocation of attentional resources (Falkenstein et al., 1994; Nieuwenhuis, Ridderinkhof, Blom, Band, & Kok, 2001). Increased Pe amplitude has been associated with improved task performance (Mathewson, Dywan, & Segalowitz, 2005), and in TS paradigms Pe amplitudes are lower for switch than for repeat trials suggesting possible task confusion (Ikeda & Hasegawa, 2012).

### The present study

The goal of the present study was to investigate the impact of targeted at-home GCCT on cognitive flexibility. Healthy young adults were randomly assigned to one of three training schedules: flexibility, attention, or control. Each training included four brain-training games, targeting task switching and cognitive flexibility for flexibility training, attention and working memory for attention training, and arithmetic for the control training. A training duration of 15 h of gameplay was pursued, in 20 sessions of 45 min over the span of 4 weeks. Such a schedule is roughly in line with optimal training duration parameters (Lampit et al., 2014), and additionally reflects an ecologically realistic estimate of at-home training times.

The games were selected from the commercially available brain-training games by Lumos Labs Inc (San Francisco, CA), and are designed to incorporate key principles for maintaining motivation during gaming (Green & Seitz, 2015; Hardy & Scanlon, 2009). For each condition at least two out of four games emulated hallmark psychological tasks for the targeted cognitive function. The active control condition was chosen to minimize extraneous confounding factors, while engaging cognitive flexibility to a lesser extent than the other training schedules. Nevertheless, questionnaires were also administered prior to and following the test sessions, to assess potentially confounding differences in expectations, motivation or enjoyment of the different trainings (Boot, Blakely & Simons, 2011). Cognitive flexibility prior to and following the training period was evaluated using the alternating-runs task-switch paradigm (Rogers & Monsell, 1995).

Firstly, for both flexibility and attention training, we expected to find overall reduced RT (and possibly ACC) as compared to control, as previous studies (e.g., Minear & Shah, 2008) have found training-induced reductions in RT on mixed blocks (which all of our blocks were). Secondly, we expected near transfer of the flexibility training, specifically, to lower local switching cost in RT (and possibly ACC). Such effects would be in line with the results found in previous training studies that incorporated task-switching exercises in the training (Karbach & Kray, 2009). Thirdly, as the flexibility training featured rapid switches between interfering rule-sets in three of the games versus zero for attention and one for control, we expected to see a specific advantage for the flexibility training in the presence of crosstalk. Greater improvements in the crosstalk blocks for the incongruent trials compared to the congruent/neutral trials would suggest improved reactive cognitive control in suppressing conflicting information. Unfortunately, with regard to time constraints we opted to not include pure-blocks in our task design, in favor of the no-crosstalk blocks (in line with Karayanidis et al., 2003), prohibiting us from evaluated mixing costs. Finally, we were interested to see whether induced benefits would be greater for longer RSI trials, indicating improved preparatory processing, or for short RSI trials (which generally show the largest switch costs).

To further dissociate such differential effects of the training schedules, we recorded EEG activity during pretest and posttest. In line with prior findings (Gajewski & Falkenstein, 2012; Gehring & Knight, 2000) as discussed above, we predicted the flexibility training in particular to result in enhanced N2 amplitude compared to the control training, indicating improved response selection. The attention training was expected to increase cognitive resources as reflected by elevated P3b amplitudes after training, especially during high-conflict trials. Furthermore, we predicted participants in the flexibility group to show enhanced Ne/ERN after training as compared to control, reflecting improved error detection. Finally, we predicted that flexibility training would show the largest decrease in Nc/CRN amplitude after training, indicating improved monitoring and stimulus–response mapping.

## Methods

### Participants

Seventy-seven participants (43 women), in majority students of Leiden University (mean age 23, range 18–37 years), were recruited without reference to experimental vs. control condition (cf. Boot et al., 2011) for €100 or €56 plus course credit. Exclusion criteria included: self-reported history of psychiatric illness, current medication use (except contraception), colorblindness and more than 30 min of video gaming per day. The study was conducted in accordance with relevant regulations and institutional guidelines (including the 1964 Declaration of Helsinki), and was approved by the ethical committee of the Institute of Psychology (Leiden University). All participants gave informed consent prior to participation.

### Design

**Fig. 1:**
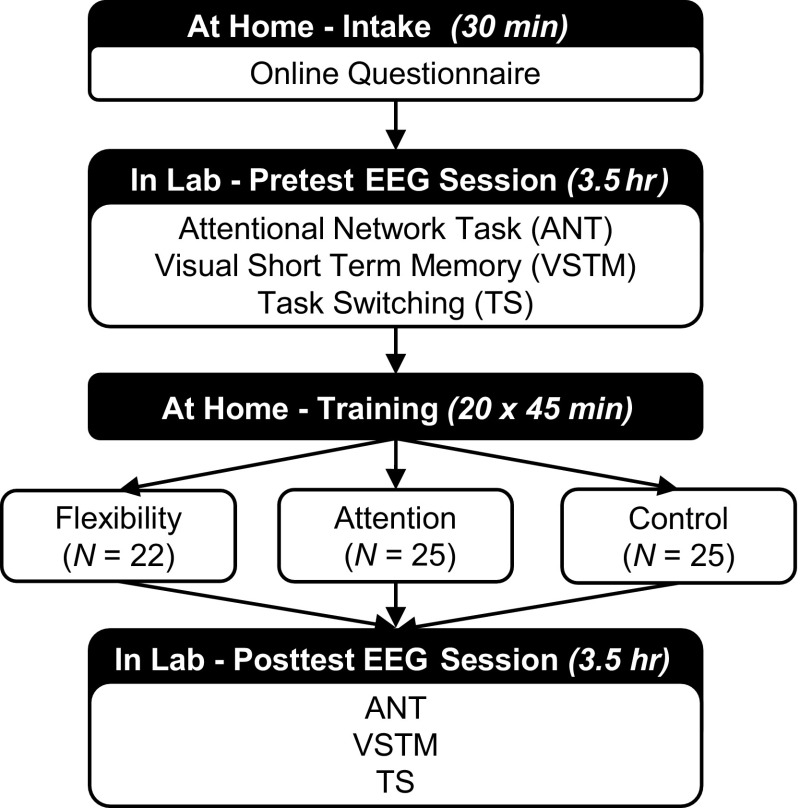
Schematic overview of the study design

The experimental design was a randomized controlled pretest–training–posttest study, as specified in Fig. 1. The main independent variable was the randomized game-training assignment: flexibility, attention, or control. Pretest and posttest measures consisted of two questionnaires and three cognitive tasks: a Task Switching paradigm (TS), the Attentional Network Task (ANT; Fan, Mccandliss, Sommer, Raz, & Posner, 2002), and the Visual Short Term Memory task (VSTM; Vogel & Machizawa, 2004). Task order and button assignment were balanced across participants. Dependent task measures included reaction times (RT) and accuracy (ACC) for the TS. Results of the ANT and VSTM are not reported in the current paper. EEG activity was measured during the entire task administration.

### Procedure

Participants completed an online lifestyle questionnaire prior to pretest, including: demographic questions; self-report of life-style habits such as time spent watching TV, using a PC, playing musical instruments or practicing sports, and gaming habits; expectations concerning the effects of the training on their brains’ functioning. Following informed consent, EEG recording was prepared and participants performed the three cognitive tasks (ANT, TS and VSTM), each lasting approximately 50 min, divided by 10–15 min breaks, total duration of the pretest session was approximately 3.5 h. Participants were comfortably seated at approximately 90 cm from a CRT or LCD monitor with 60 Hz refresh rates. Visual angles of stimuli were held constant across setups. Responses were made on two Serial Response Boxes (Psychology Software Tools, Inc.). After completion of the training (15 sessions or more), participants returned to the lab for a posttest session. The posttest session mirrored the pretest, but concluded with a questionnaire concerning the experience, motivation and expectations during training, and a debriefing.

### Game training

The game training included four online brain-training games per group, targeting: (1) task switching and cognitive flexibility for the flexibility group, (2) attention and working memory for the attention group, and (3) arithmetic for the control group. All games: required fast and accurate responses to gain the most points, featured adaptive difficulty and had a fixed time-limit or a fixed amount of ‘lives’ (allowed errors). The games were provided on Lumosity.com, from Lumos Labs Inc (San Francisco, CA), for which participants were given a personal account. Participants were asked to play 20 sessions of 45 min (15 h in total), at home or wherever they had access to a pc or laptop (with an external mouse). Participants played five sessions per week at approximately the same time each day, and were free to choose which days they played. Daily progress reports were monitored and participants were reminded if they failed to follow the assigned game schedule. Missed or incomplete training session could be compensated during one of the following days. Training was considered completed if at least 15 sessions had been played prior to posttest.

We chose four different games per training partly to prevent boredom and maintain similar training structures, rather than the availability of games that precisely targeted the intended cognitive functions. Furthermore, all games likely challenged multiple cognitive functions to some extent (e.g., attention and working memory can be assumed to be important for most speeded response tasks). Crucially though, each training featured at least two games that emulated hallmark psychological tasks for the targeted function which have shown positive effects in prior (G)CCT studies. Specifically, the flexibility training contained two games highly similar to the standard task-switching paradigm (Karbach & Kray, 2009; Rogers & Monsell, 1995). Likewise, the attention and working memory training included games resembling the Useful Field of View (Ball, Beard, Roenker, Miller, & Griggs, 1988), and N-back task (Jaeggi et al., 2010; Kirchner, 1958). Moreover, the flexibility training featured the most instances of prototypical task switching: within the games a rapid change in interfering rule-sets between trials/turns was present in three games for the flexibility training, zero games for the attention and one game for control. All groups switched between the games equally within and between sessions. A full description of each game falls outside the current scope; however, brief descriptions are provided below.

### Flexibility

*Brain Shift Overdrive* similar to the classic task-switching paradigm (Rogers & Monsell, 1995), digit-letter pairs were presented in a 2 × 2 grid, each position associated with a specified task (e.g., indicating if the letter is a vowel or the digit is even). Notably, repeat and switch trials were presented in randomized order (though equal in number), rather than in alternating fashion. *Disillusion* Players cleared puzzle boards by connecting new pieces, matching either the color or the symbol on the pieces depending on its orientation, the latter switched randomly between pieces/turns. *Penguin Pursuit* Players navigate a penguin through a maze. At random intervals, the maze rotated 90 or 180 degrees and the button mapping rotated accordingly (e.g., the ‘up’ key would correspond to a left-movement after a counterclockwise 90° turn). *Rotation Matrix* Participants had to remember the position of colored blocks that briefly appeared on a grid, and indicate them after the grid rotated 90° or 180°.

### Attention

*Eagle Eye* resembled the useful field of view task (Ball et al., 1988). Participants reported the number briefly flashed in the center of the screen and the location of a target (bird) that was presented concurrently in the periphery, while ignoring distractors. *Playing Koi* is an object tracking game: players had to click on multiple fish moving randomly across the screen, without clicking the same fish twice and with a forced delay between each click. *Monster Garden* showed participants a target on a garden grid. Obstacles were briefly presented, after which the player had to navigate towards the target while avoiding the now invisible obstacles. Memory match overload is a visual N-back task (Jaeggi et al., 2010; Kirchner, 1958): sequences of fruits were shown, and participants indicated whether the currently shown fruit was equal to that shown *N* items prior.

### Control

*Multiplication Storm*,* Division Storm* and* Subtraction Storm* multiple math problems were concurrently falling to the bottom of the screen (at varying speeds) requiring the participant to solve them using the titular calculations before they disappeared.* Rain Drops*: similar to the first three games, except the math problems featured any of the three types of calculations.

### Task-switching paradigm

**Fig. 2:**
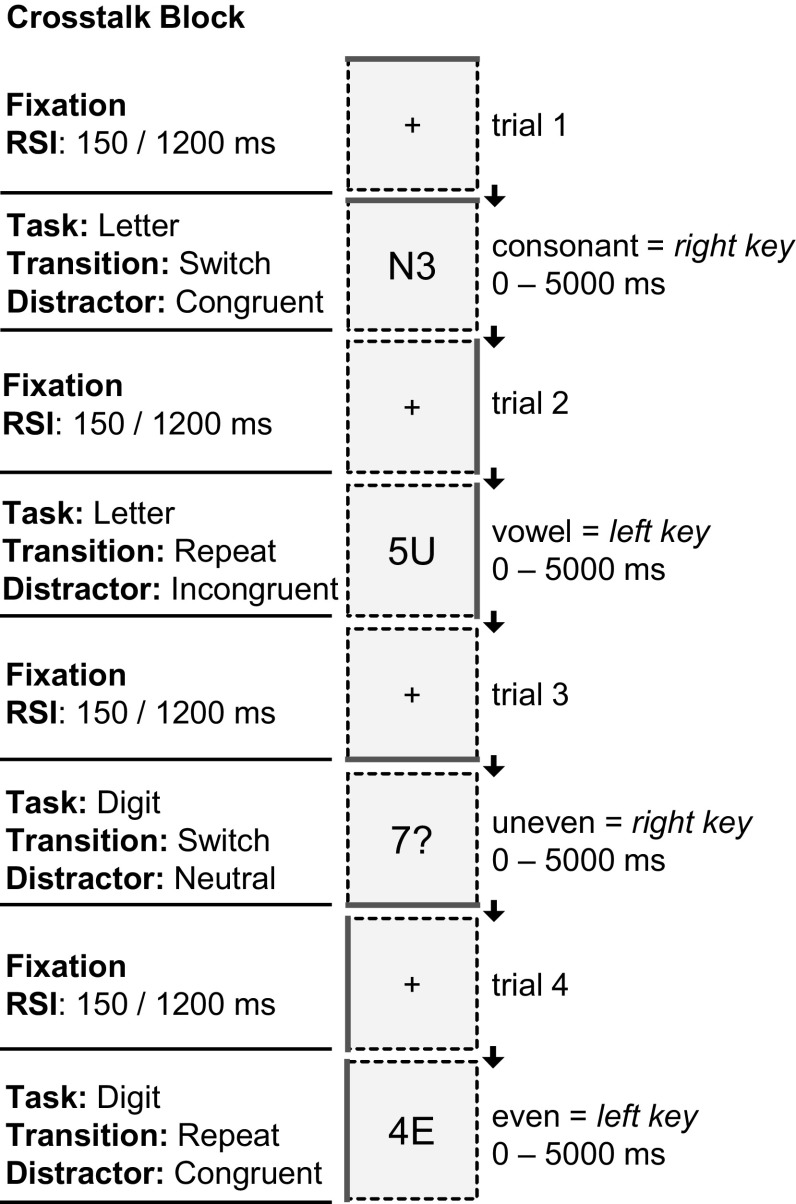
Example trials of the TS for crosstalk blocks. The RSI conditions varied between but not within blocks. The no-crosstalk blocks (not shown here) only featured neutral distractors

The alternating-runs TS was adapted from Karayanidis and colleagues (2003), and is illustrated in Fig. 2. Participants performed two tasks: a letter task and a digit task, task type switched predictably every second trial (AABB). For the letter task participants classified the target as a vowel (A, E, I, U) or a consonant (G, K, M, R), and for the digit task as even (2, 4, 6, 8) or odd (3, 5, 7, 9), using the right or left response buttons with their respective index fingers (counterbalanced across participants). Stimuli always consisted of one task-relevant character (a letter or digit) and one task-irrelevant distractor character (letter, digit or non-alphanumeric). The distractor could be associated with the same (congruent) or opposite response (incongruent) in the context of the currently irrelevant task, or be neutral, as in the case of trials with non-alphanumeric distractors (#, ?, *, %). Stimuli were presented until participants made a button press or for a total of 5000 ms. The blockwise varying response–stimulus interval (RSI) could have a length of 150 ms or 1200 ms. So-called crosstalk blocks contained 1/3 congruent, 1/3 incongruent and 1/3 neutral trials, whereas no-crosstalk blocks only contained neutral trials, yielding four block types: crosstalk 150 ms, crosstalk 1200 ms, no-crosstalk 150 ms and no-crosstalk 1200 ms.

After four training blocks, participants were instructed to respond as quickly and as accurately as possible and the four test block types were presented (order randomized over participants, but kept equal between pretest and posttest). Each block type was presented for 4 consecutive runs. Every run featured 96 randomized test trials (preceded by 4 buffer trials). In total there were 64 trials for each combination: RSI (150/1200) × Transition (switch/repeat) × Distractor (neutral/congruent/incongruent) in the crosstalk blocks, and 192 trials for each combination of RSI × Transition in the no-crosstalk blocks. After each run, performance feedback was given and participants could take a short break.

Contrary to Karayanidis and colleagues (2003), stimuli were not presented in a 2 × 2 grid. Instead, to minimize eye-movement related EEG artifacts and location-based confound of performance (Arbuthnott, Woodward, & Columbia, 2002), all imperative stimuli were presented (in Arial 20 point font) in the center of a square blue square outline (10 cm × 10 cm, visual angle *θ* = 6.36°), on a light grey background (rgb: 192, 192, 192). The current task type was indicated by making one of the sides of the box magenta colored (top, right, bottom, or left), rotating clockwise every trial. The mapping of box side to task type was balanced between participants.

### Analysis

Mean RT was calculated for correct responses, for each combination of: Session (pretest/posttest) × Block (no-crosstalk/crosstalk) × Task × RSI × Transition × Distractor. Responses outside a 100–4000 ms range were recorded as misses (no response), as well as responses more than 3 standard deviations away from the cell mean of each participant and trials following incorrect responses. Accuracy (ACC) as expressed in error score proportions, were arcsine transformed for analysis, although untransformed percentages are numerically reported for interpretation.

All analyses were run separately for RT and for ACC, and for the no-crosstalk and crosstalk blocks as these contained different trial conditions (in line with Karayanidis et al., 2003). For the no-crosstalk block, the ANOVA included within-subject factors Session, Type, RSI, and the between-subject factor Group. For the crosstalk blocks, the ANOVA additionally included the within-subject factor Distractor. Where appropriate, Greenhouse–Geisser adjusted values were used as correction for violations of the sphericity assumption (Vasey & Thayer, 1987). The significance level was set at α ≤ 0.05, and partial eta squared (*η*_*p*_^2^) is reported as an estimate of effect size. In line with our hypotheses, we focused on the interactions: Session × Group for overall performance; Session × Group × Transition for training effects on local switch costs (calculated by subtracting the mean performance on the repeat trials from the switch trials); and Session × Group × Distractor for crosstalk congruency effects. Significant interactions in these cases were evaluated with a priori contrasts, comparing the posttest–pretest gain-scores for: (1) flexibility versus control, (2) attention versus control, (3) flexibility versus attention. Interaction effects outside this scope but involving Group × Session (including for instance RSI) were followed up post hoc with exploratory Bonferroni-corrected pairwise comparisons.

### EEG data acquisition and analysis

Electroencephalographic (EEG) activity was measured with active BioSemi electrodes over 32 positions as defined in the 10-10 system: Fp1, Fpz, Fp2, F7, F3, Fz, F4, F8, FC3, FCz, FC4, C3, Cz, C4, T7, T8, CPz, TP7, TP8, P7, P3, Pz, P4, P8, PO7, PO3, POz, PO4, PO8, O1, Oz, and O2. Horizontal eye movements were calculated by bipolar derivations of electro-oculogram (EOG) signals over the left and right outer canthus. Vertical eye movements were calculated by bipolar derivations of signals above and below the right eye. Monopolar recordings were referenced to the common mode sensor (CMS) and drift was corrected with a driven right leg (DRL) electrode (for details see http://www.biosemi.com/faq/cms&drl.htm). Offline analyses were performed with Brain Vision Analyzer. Due to a malfunction in the mastoid electrodes, data were re-referenced offline to the T7 and T8 positions, or in case of excessive noise on those channels to TP7 and TP8. Data were high-pass filtered at 0.05 Hz (24 dB/oct) to effectively remove drift. Ocular artifacts were corrected using the regression approach (Gratton, Coles, & Donchin, 1983). Trials with movement artifacts were rejected, and remaining data were low-pass filtered at 15 Hz (24 dB/oct). Stimulus and response-locked average waveforms per condition were computed from artifact-free segments: on average 177 out of 192 (SD = 13) segments for the no-crosstalk block conditions and 58 out of 64 (SD = 5) segments per condition for the crosstalk blocks.

The electrode sites were chosen from one of the midline electrodes (Fz, FCz, Cz, CPz or Pz), after visual inspection of the grand-averaged ERPs collapsed over participants, conditions and sessions. ERP components were computed similar to Gajewski and Falkenstein (2012). Target-locked waveforms were baseline-corrected for the 100 ms pre-target interval. N2 was evaluated at FCz as the negative local peak 200–500 ms post-target; P3b at CPz as the positive local peak 350–700 ms post-target. Response-locked waveforms were baseline-corrected for the 100 ms pre-response interval. The Ne/ERN was quantified for incorrect responses, as the negative going peak at FCz 0–150 ms post-response. The Nc/CRN was evaluated for correct responses, as the most negative local peak at Fz 0–150 post-response. The Pe was evaluated for incorrect responses, as the positive going peak at FCz 50–250 ms post-response. All ERP components were analyzed with ANOVA designs and prepared contrasts equivalent to the behavioral analysis.

## Results

### Sample descriptives

The data of 72 participants were analyzed (flexibility *N* = 22, attention *N* = 25, control *N* = 25), after dropout (2) and exclusion due to medical condition (1), inability to complete the task within the time-limits (1) and technical error (1). Bonferroni-corrected* t* tests of the questionnaire data revealed no group difference on: mean age, gender, nationality, completed level of education, or time on leisure time activities (including gaming, sports or playing music instruments), enjoyment of the training, motivation for participation, prior expectations about brain-training effects, expectations of brain-training effects after the posttest, or the change in expectations, all *p* > 0.3. The training groups did not differ on estimated training time: flexibility group 14.0 h (SD = 1.6), attention 13.5 h (SD = 1.8) and control group 14.2 h (SD = 2.2), *p* = 0.377, nor on average time between pretest and posttest (*M* = 34 days), *p* > 0.5.

### Behavioral effects

Four participants were excluded from further analysis: one showed an abnormally large increase in reaction time after training (> 2.6 *SD* from group mean) and three participants had insufficient useable segments in the EEG signal (less than 75% of total trials), leaving 68 participants (flexibility *N* = 21, attention *N* = 25, control *N* = 22). While a full description of all TS interaction effects falls outside the scope of this paper, the stereotypical effects (pooled across training conditions and sessions) followed the patterns observed in comparable studies (e.g., Karayanidis et al., 2003). Below we describe the results relating to our hypotheses: Session × Group interactions (for general task improvement) including Transition (for local switch costs effects), Distractor (for local crosstalk effects) and RSI (for preparation costs). The relevant ANOVA statistics can be found in Table 1, while RT and error percentages are shown per factor of interest in Table 2. Notably, ANOVA of pretest RT and ACC showed no main Group effect, *p* > 0.6.

**Table 1:** Task-switch ANOVA statistics for mean RT and arcsine transformed error proportions, separately for no-crosstalk (NC) and crosstalk (C)

ANOVA effects	Block	RT				Arcsine error %			
		*(df)*	*F*	*p*	*η*_*p*_^*2*^	*(df)*	*F*	*p*	*η*_*p*_^*2*^
Session	NC	(1,65)	82.07	< 0.001**	0.558	(1,65)	3.71	0.058	0.054
	C	(1,65)	133.58	< 0.001**	0.673	(1,65)	4.94	0.030*	0.071
Group	NC	(2,65)	0.33	0.723	0.010	(2,65)	1.68	0.195	0.049
	C	(2,65)	0.30	0.743	0.009	(2,65)	0.18	0.839	0.005
Group × Session	NC	(2,65)	3.21	0.047*	0.090	(2,65)	1.91	0.157	0.055
	C	(2,65)	3.35	0.041*	0.093	(2,65)	0.90	0.411	0.027
Transition	NC	(1,65)	140.26	< 0.001**	0.683	(1,65)	75.63	< 0.001**	0.538
	C	(1,65)	255.14	< 0.001**	0.797	(1,65)	134.89	< 0.001**	0.675
Transition × Session	NC	(1,65)	50.74	< 0.001**	0.438	(1,65)	31.34	< 0.001**	0.325
	C	(1,65)	71.78	< 0.001**	0.525	(1,65)	5.06	0.028*	0.072
Transition × Session × Group	NC	(2,65)	1.37	0.262	0.040	(2,65)	0.11	0.898	0.003
	C	(2,65)	0.17	0.847	0.005	(2,65)	0.93	0.400	0.028
Distractor	C	(2,130)^a^	112.64	< 0.001**	0.634	(2,130)^c^	126.29	< 0.001**	0.660
Distractor × Session	C	(2,130)^b^	13.13	< 0.001**	0.168	(2,130)^d^	0.24	0.721	0.004
Distractor × Session × Group	C	(4,130)^b^	3.20	0.028*	0.085	(4,130)^d^	0.48	0.751	0.015

Greenhouse–Geisser corrected at ^a^ *ɛ* = 0.592, ^b^ *ɛ* = 0.838, ^c^ *ɛ* = 0.660, ^d^ *ɛ* = 0.746

Significant at * *α* ≤ 0.05 and ** *α* ≤ 0.001

**Table 2:** Average RT and error percentage scores per group for pretest (S1) and posttest (S2) sessions

	RT			Error %		
	Flexibility	Attention	Control	Flexibility	Attention	Control
	*M* (SE)	*M* (SE)	*M* (SE)	*M* (SE)	*M* (SE)	*M* (SE)
No-crosstalk						
Overall						
S1	714 (37)	738 (34)	669 (36)	4.7 (0.6)	4.2 (0.6)	3.3 (0.6)
S2	590 (25)	591 (23)	597 (25)	3.7 (0.6)	4.1 (0.5)	2.8 (0.5)
Repeat						
S1	608 (26)	637 (24)	587 (25)	3.1 (0.5)	2.6 (0.4)	2.2 (0.5)
S2	521 (18)	536 (17)	542 (18)	2.9 (0.5)	3.1 (0.4)	2.3 (0.5)
Switch						
S1	820 (49)	839 (45)	750 (48)	6.2 (0.9)	5.8 (0.8)	4.5 (0.8)
S2	659 (35)	646 (32)	653 (34)	4.4 (0.7)	5.1 (0.7)	3.3 (0.7)
Crosstalk						
Overall						
S1	980 (58)	999 (53)	916 (57)	5.2 (0.8)	4.7 (0.7)	4.7 (0.8)
S2	742 (44)	796 (40)	783 (43)	4.1 (0.6)	4.4 (0.6)	3.9 (0.6)
Repeat						
S1	839 (53)	835 (48)	772 (52)	3.7 (0.9)	3.1 (0.8)	3.5 (0.8)
S2	638 (37)	675 (34)	683 (36)	3.4 (0.6)	3.0 (0.6)	2.9 (0.6)
Switch						
S1	1120 (66)	1164 (60)	101 (65)	6.7 (0.8)	6.1 (0.7)	5.8 (0.9)
S2	845 (54)	918 (50)	884 (52)	4.8 (0.8)	5.9 (0.7)	5.0 (0.7)
Neutral						
S1	828 (44)	857 (40)	797 (42)	3.1 (0.5)	2.8 (0.5)	2.7 (0.5)
S2	650 (34)	679 (31)	673 (33)	2.1 (0.4)	2.5 (0.3)	1.8 (0.4)
Congruent						
S1	1046 (65)	1064 (59)	962 (63)	1.0 (0.6)	3.4 (0.5)	2.8 (0.6)
S2	780 (50)	842 (46)	825 (49)	2.7 (0.6)	3.1 (0.5)	2.5 (0.5)
Incongruent						
S1	1065 (71)	1077 (65)	990 (69)	8.4 (1.6)	7.8 (1.4)	8.5 (1.5)
S2	795 (50)	869 (46)	852 (49)	7.5 (1.3)	7.7 (1.2)	7.6 (1.2)

### General performance

**Fig. 3:**
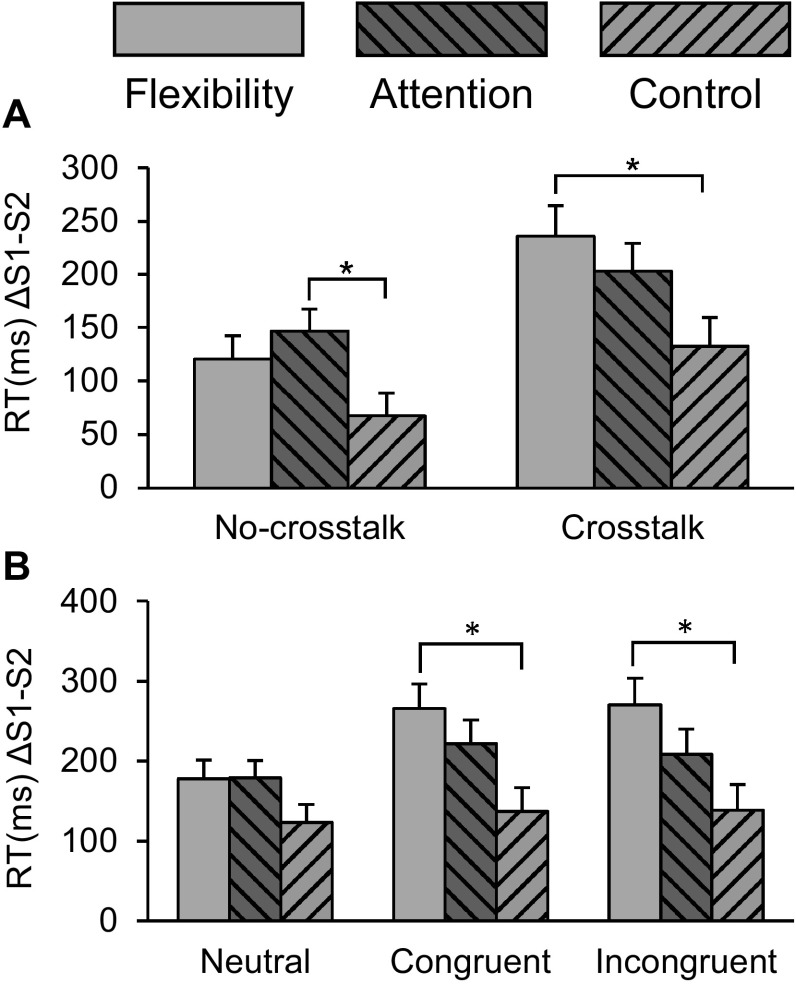
Changes in RT (pretest-posttest) per training group (**a**) separate for no-crosstalk and crosstalk, collapsed over all task conditions (**b**) for crosstalk, separate for each distractor condition and collapsed over all other task conditions. All RTs improved significantly at *p* < 0.005. Error bars denote SE. * p < 0.05

Our first prediction was that both flexibility and attention training would induce benefits in overall performance as compared to control. A main effect of Session was found for both crosstalk and no-crosstalk blocks: RTs were longer at pretest than at posttest. Importantly, a significant Group × Session interaction was present for both block types, with planned comparisons indicating decreased RT after training for all groups (*p* < 0.001). For no-crosstalk, prepared contrasts showed greater improvement for the attention group compared to the control group (*p* = 0.015), while for the crosstalk blocks a significantly greater improvement was present for flexibility as compared to the control group (*p* = 0.014); see Fig. 3a.

### Local switch costs

Our second prediction was that flexibility training would selectively decrease local switch costs after training. ANOVA of RT performance showed a main effect of Transition (switch/repeat) for both no-crosstalk and crosstalk blocks, with shorter RTs on repeat compared to switch trials, indicating reliable switch costs. Both blocks also showed a Transition × Session interaction: the decrease in RTs was significant for both repeat and switch trials, but larger for the latter, as indicated by significantly smaller switch costs, all *p* < 0.001. Planned comparisons indicated lower switch costs for all groups after training, for both crosstalk and no-crosstalk blocks, all *p* < 0.05. However, no significant interactions including Transition × Session × Group were found.

### Crosstalk effects

Our third prediction was that the flexibility group would have an advantage in the presence of distractor-induced crosstalk. ANOVA of RT performance in the crosstalk blocks showed a main effect of Distractor: RTs were significantly shorter on neutral (*M* = 747, SD = 21) than RTs on congruent trials (*M* = 920, SD = 31) which in turn were shorter than RTs on incongruent trials (*M* = 941, SD = 32), all at *p* < 0.01. There was also an interaction of Distractor × Session: RTs decreased for all distractors (*p* > 0.001), but significantly less for neutral trials (Δ*M* = −160, SE = 13) than either congruent (Δ*M* = −208, SE = 18) or incongruent trials (Δ*M* = −206, SE = 20), *p* < 0.005. Importantly, there was a three-way interaction of Session × Group × Distractor. As illustrated in Fig. 3b, all groups improved for each distractor condition, all *p* < 0.001. However, a priori contrasts between the groups revealed that only flexibility improved significantly more compared to control, for both congruent and incongruent distractors, all *p* < 0.05. The flexibility group improved more for both congruent and incongruent trials than for neutral trials, all *p* < 0.001. For the attention group, only performance on congruent trials improved significantly more than for neutral trials, *p* = 0.009. The control group showed no differences in change scores between any of the distractors.

### Accuracy

Error performance was analyzed similar to the RT effects above, but revealed no significant interaction effects including Group × Session; see Table 2 for relevant ANOVA statistics.

### ERP effects

ERP analysis included the stimulus-locked N2 and P3b, and the response-locked Nc/CRN and Ne/ERN. Below we describe per component significant results relating to our hypotheses: Session × Group interactions (for general task improvement) including Transition (for local switch costs effects), Distractor (for local crosstalk effects) and RSI (for preparation costs).

### N2 component

**Fig. 4:**
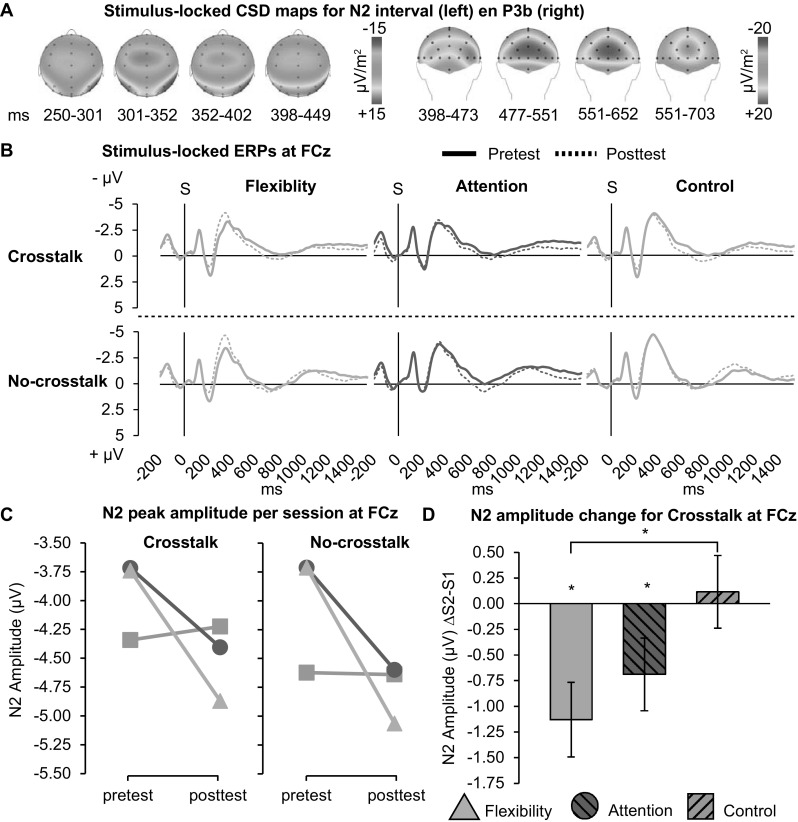
Stimulus-locked ERPs. **a** Grand average CSD maps for N2 and P3b intervals. **b** ERPs at FCz, per Group and Block, for pretest (solid lines) and posttest (dashes lines), vertical lines indicate stimulus onset. **c** Average N2 peak amplitudes at FCz, per group and session. **d** N2 peak amplitude change (posttest–pretest) per group for crosstalk only. Error bars denote SE. * p ≤ 0.05

N2 peak amplitude was evaluated at Cz; see Fig. 4a for scalp voltage maps and Fig. 4b for the stimulus-locked ERPs per group and session. There was a main effect of Session for crosstalk, *F*(1,65) = 7.88, *p* = 0.007, *η*_*p*_^2^ = 0.108, and no-crosstalk blocks, *F*(1,65) = 6.01, *p* = 0.017, *η*_*p*_^2^ = 0.085, N2 amplitude increased after training for both blocks. Importantly, there was a (barely) significant interaction of Group × Session for the crosstalk block, *F*(2,65) = 3.12, *p* = 0.051, *η*_*p*_^2^ = 0.088. As illustrated in Fig. 4b, both flexibility (*p* = 0.003) and attention (*p* = 0.042) showed a significant increase in N2, while control did not. Furthermore, prepared contrast revealed that only for flexibility N2 increased significantly more as compared to control (*p* = 0.017). No main effects of Group were present at pretest (*p* > 0.562). The no-crosstalk block showed a similar but non-significant trend for Session × Group (*p* = 0.062). In contrast to our predictions regarding local switch costs and crosstalk effects, no other interaction effects including Group × Session were found for either block (all *p* > 0.208).^1^

### P3 component

P3b was evaluated at CPz; see Fig. 4a for scalp voltage maps. ANOVA of P3b peak amplitude revealed no effect of Session for crosstalk block (*p* = 0.110), or no-crosstalk block (*p* = 0.783). Despite our predictions, no interactions including Session × Group were found, all *p* > 0.2.

### Nc/CRN component

**Fig. 5:**
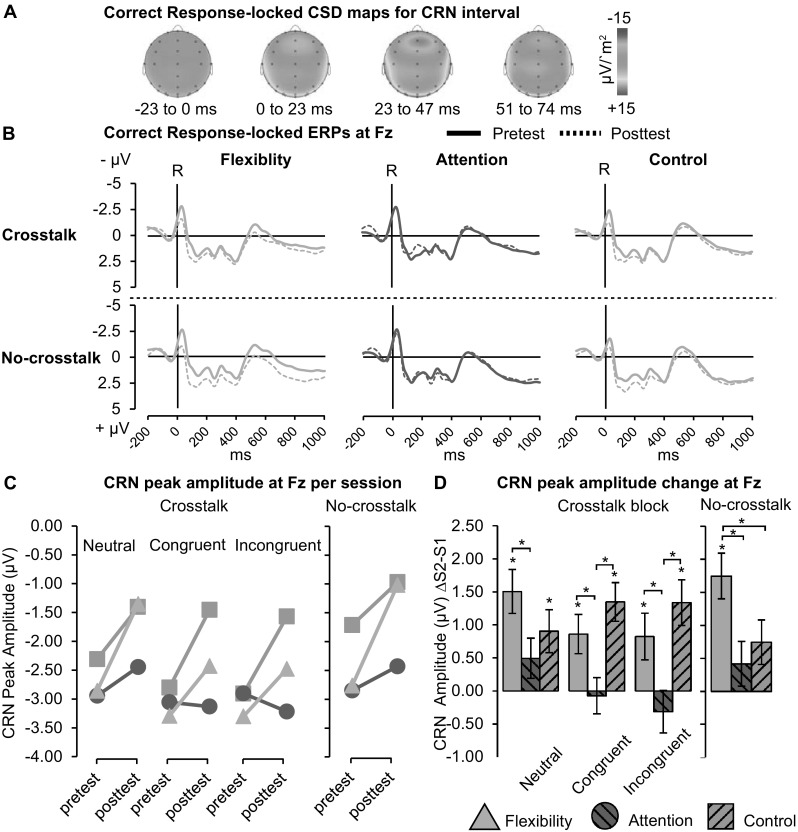
Correct response-locked ERPs. **a** Grand average CSD maps for CRN interval. **b** ERPs at Fz, per group, for pretest (solid lines) and posttest (dashes lines), vertical lines indicate response onset. **c** Average CRN peak amplitudes, per group, session, block and distractor. **d** CRN peak amplitude change (posttest–pretest) per group and distractor, for crosstalk only. Error bars denote SE. * *p* ≤ 0.05

Nc/CRN was evaluated at Fz; see Fig. 5a for scalp voltage maps and Fig. 5b for the response-locked ERPs per group and session. A main effect of Session was found for both crosstalk, *F*(1,65) = 23.57, *p* < 0.001, *η*_*p*_^2^ = 0.266, and no-crosstalk, *F*(1,65) = 25.15, *p* < 0.001, *η*_*p*_^2^ = 0.279, with Nc/CRN amplitude decreasing after training. Partially corresponding to our hypothesis, a Group × Session interaction was found for no-crosstalk, *F*(2,65) = 3.60, *p* = 0.019, *η*_*p*_^2^ = 0.115, and crosstalk blocks, *F*(1,65) = 5.70, *p* = 0.005, *η*_*p*_^2^ = 0.149. Planned comparisons revealed a significant decrease in Nc/CRN amplitude in both blocks after flexibility and control training (*p* < 0.05), but not for attention (*p* > 0.1); see Fig. 5c. For the crosstalk blocks both flexibility and control Nc/CRN decreased more than for attention (*p* < 0.01), while for the no-crosstalk blocks flexibility showed a larger reduction compared to both attention (*p* = 0.006) and control (*p* = 0.042). Importantly, there was no main effect of Group at pretest for either block type (*p* ≥ 0.16).

Furthermore, the crosstalk blocks revealed a Group × Session × Distractor effect, *F*(4130) = 3.60, *p* = 0.008, *η*_*p*_^2^ = 0.100. Planned comparisons revealed significant reductions in Nc/CRN amplitude after flexibility and control training for all distractor conditions (*p* < 0.05), but none for attention (*p* > 0.1). As indicated in Fig. 5d, for congruent and incongruent distractors both flexibility and control Nc/CRN decreased more than attention, for neutral trials only flexibility decreased more than attention, all *p* < 0.05. Importantly, there was no Group × Distractor effect at pretest, *p* = 0.310. Finally, exploratory pairwise Bonferroni-corrected comparisons of the Nc/CRN amplitudes between the distractors at posttest, revealed that for flexibility and attention Nc/CRN amplitude was significantly smaller for the neutral trials as compared to either congruent or incongruent trials (*p* < 0.05), while the control group showed no such differences (*p* > 0.99).

### Ne/ERN component

**Fig. 6:**
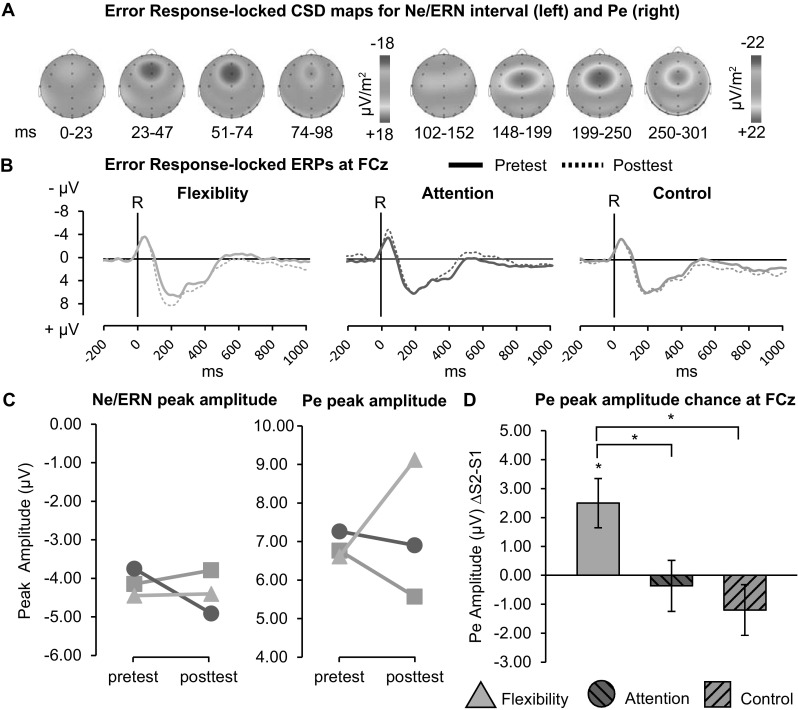
Error response-locked ERPs. **a** Grand average CSD maps for Ne/ERN and Pe intervals. **b** ERPs at FCz, per group, for pretest (solid lines) and posttest (dashes lines), vertical lines indicate response onset. **c** Average Ne/ERN and Pe peak amplitudes, per group and session. **d** Pe peak amplitude change (posttest–pretest) per group for crosstalk only. Error bars denote SE. * p ≤ 0.05

Ne/ERN was assessed at FCz; see Fig. 6a for scalp voltage maps and Fig. 6b for the response-locked ERPs per group and session. Full factorial ANOVAs of Ne/ERN amplitudes were not possible, as there were too few participants with error segments in all task conditions. Instead, we evaluated Ne/ERN over all incorrect trials for participants with EEG data on at least 20 error trials (*N*_*p*_ = 57), with an average of 75 (SD = 44) segments per participants in the pretest and 67 (SD = 38) in the posttest. One-sample t-tests showed robust Ne/ERN peak amplitude for pretest (*M* = −4.08 μV, SD = 2.37) and posttest (*M* = −4.42 μV, SD = 2.63), both *p* < 0.001. In contrast to our prediction, Ne/ERN did not increase after training as ANOVAs revealed no Session or Session × Group effects, (*p* > 0.139); see Fig. 6c.^2^

The error response-locked grand-average ERP seemed to indicate a strong error positivity (Pe); see Fig. 6a. Therefore, we conducted an additional exploratory ANOVA on Pe peak amplitude at FCz (as the most positive local peak 50–250 ms post-response), using the same criteria as for Ne/ERN. One-sample *t* test showed robust Pe for both pretest (*M* = −6.91 μV, SD = 3.22) and posttest (*M* = −7.21 μV, SD = 4.10), both* p* < 0.001. ANOVA revealed no main effect of Session, *F*(1,54) = 0.43, *p* = 0.516, *η*_*p*_^2^ = 0.008. Importantly, there was a Group × Session interaction, *F*(2,54) = 5.2, *p* = 0.009, *η*_*p*_^2^ = 0.160. Bonferroni-corrected post hoc comparisons indicated only flexibility training yielded a significant increase in Pe amplitude (*p* = 0.005), moreover this increase was significantly greater than for attention (*p* = 0.048) or control (*p* = 0.011); see Fig. 6c and d. Notably, there was no main effect of Group at pretest, *F*(2,65) = 0.16, *p* = 0.849, *η*_*p*_^2^ = 0.005.

## Discussion

In the current study we investigated whether game-based computerized cognitive training (GCCT) could enhance cognitive flexibility in healthy young adults. Specifically, the question was if GCCT would be effective when explicitly targeted at cognitive flexibility (near-transfer) and if a more general training (i.e., not targeting cognitive flexibility) would also result in task-switch performance benefits (far-transfer). Three GCCT schedules were contrasted, targeting: (1) cognitive flexibility and task switching in particular (2) attention and working memory, or (3) an active control (involving math games). Performance on an alternating-runs task-switch paradigm (TS) was tested before and after a 15-h training to assess transfer effects. Additionally, event-related potentials (ERPs) were recorded during pretest and posttest sessions, to elucidate the neural mechanisms underlying any induced changes behavioral performance.

### General performance

As predicted, flexibility training led to greater improvement in general reaction times (RT) after training, as compared to the control group. Additionally, the attention training also induced greater improvement in RT compared to the control. Given the targeted nature of the GCCTs, these improvements constitute near-transfer and far-transfer, respectively. Notably, the near- and far-transfer effects were found under different task conditions. The near-transfer RT effect of the flexibility training was present only during crosstalk blocks (which included trials with distractors from the irrelevant task-sets). In line with Gajewski and Falkenstein, (2012), behavioral results were mirrored by an increase in fronto-central N2 amplitude, with a larger N2 increase observed specifically after flexibility training as compared to control. N2 has been related to selecting the appropriate response to a target stimulus, as determined by the relevant task rule (Gajewski et al., 2010; Swainson et al., 2003). These findings suggest that our flexibility training led to better response selection in the presence of conflicting rule-sets, rather than to rapid switching between rule-sets in general. We will explore this notion further in the “Crosstalk effects” section below. The N2 amplitude did not differentiate between flexibility and attention training, again suggesting that the latter also led to some degree of improved processing.

The far-transfer effect on general RT for the attention training was only present during the no-crosstalk blocks, which included only neutral distractor trials. One explanation for this particular benefit of attention training is that these blocks are overall less challenging, making it more difficult to maintain selective attention. Strobach and colleagues (2012) used a similar reasoning to explain why more errors tend to be made during the single-task blocks as compared to repeat trials in mixed-task blocks. Thus, the attention training may have boosted (sustained) selective attention, similar to effects found for action video game training (Karle et al., 2010). Unfortunately, the ERP data did not provide unequivocal support for this interpretation, as the attention-related P3 component did not show the expected intervention specific differences after training.

### Local switch costs

In contrast to our prediction and previous findings (Karbach & Kray, 2009), flexibility training did not lead to specific local switch cost reductions in RT or accuracy, or changes in switch-related ERP components. This absence was somewhat surprising, since the flexibility training included games that were highly similar to the task-switching paradigm used for testing. Additionally, three out of four flexibility games featured substantially more instances of switching between interfering rule-sets, compared to the other trainings. However, Pereg and colleagues (2013) found that transfer effects to local switch costs disappeared when the training and the testing paradigms contained even minor variations in task structure. The authors suggested that local-switch cost improvements might therefore be driven by task structure-specific memory effects. As the task-switch games in our study were structurally dissimilar from the testing paradigm (i.e., randomized instead of alternating task order), this might explain the current lack of transfer effects for local switch costs. Furthermore, the switches were uncued meaning the participants did not practice preparing for upcoming switches, which could also explain why training did not engender a better utilization of the long (versus short) response–stimulus intervals for preparatory processing.

Despite the above, the notion that improvements on local switch costs are only found when the training and testing paradigm are virtually identical is contested by findings from action video game (AVG) studies (Green, Sugarman, Medford, Klobusicky, & Bavelier, 2012; Strobach, Frensch & Schubert, 2012; Wang et al., 2016). Presumably the task structure during AVGs is even further removed from the testing paradigms, as compared to cognitive training. One explanation for this disparity in findings is that AVGs often include many different types of tasks that have to be switched between frequently, whereas most cognitive training games have only one or (like the flexibility games used here) two interfering rule-sets or goals. The importance of task diversity and complexity is emphasized in the learning-to-learn theory proposed by Bavelier, Green, Pouget, and Schrater (2012), and in the benefits of multitask training as found by Anguera and colleagues (2013). An important avenue for future research could therefore be to approximate AVGs more closely, by increasing the number of overlapping concurrent tasks or rule-sets per training game even further, as well as the number of different games within a training.

### Crosstalk effects

Although more than two conflicting rule-sets or tasks might be preferable for training, the current training did induce specific performance benefits in the presence of response conflict. As mentioned earlier, the near-transfer RT effect of the flexibility training was present only during crosstalk blocks. Specifically, the improvement over the control group was selective to the trials with either congruent or incongruent distractors, and absent for the neutral distractor trials. This distractor-specific effect cannot simply be attributed to ceiling performance on neutral distractor trials, given the presence of training effects for the no-crosstalk blocks, which included only neutral trials and yielded even shorter overall RTs. Given the pattern of results for the crosstalk blocks, the flexibility training provided a specific advantage in the presence of non-neutral distractors, such that conflicting stimulus information was more successfully suppressed or ignored during response selection.

Of the ERP components, only the fronto-central correct-response-locked negativity (Nc/CRN, Vidal et al., 2003) revealed crosstalk-related training effects. The Nc/CRN is an indication of perceived conflict even when the correct response is made, with higher amplitudes reflecting suboptimal monitoring and stimulus–response mapping (Eppinger et al., 2007; Gehring & Knight, 2000). In line with our expectations, Nc/CRN decreased more after flexibility training as compared to either other training groups during no-crosstalk blocks (containing only neutral distractor trials). Similarly, in the crosstalk blocks, flexibility had a specific advantage in the neutral distractor trials as compared to attention training. Interestingly, however, for the congruent and incongruent distractor trials both the flexibility and the control group showed larger reductions in Nc/CRN compared to attention. One explanation might be that a non-discriminate decrease in Nc/CRN might not be particularly indicative of improved monitoring, as higher Nc/CRN during difficult trials as opposed to easy trials is generally regarded as a sign of efficient monitoring (Eppinger et al., 2007). Indeed, exploratory analysis revealed that while such a pattern was present at posttest for both flexibility and attention groups, the control group showed no Nc/CRN differences between the distractors. In sum, the Nc/CRN results suggest increased efficiency in response-conflict monitoring at least after flexibility training.

### Limitations and future directions

The current study did feature some limitations. First, as the current task design did not incorporate pure blocks (without switch trials), we could not investigate mixing costs on behavioral performance or ERP measures directly. In hindsight this has been an unfortunate omission. Although at least the presence of training effects of general RT does not rule out the presence of mixing cost effects, and we propose that any GCCT-specific effects on TS performance are important from an ecological viewpoint. Second, it is fair to note that by design both the flexibility and attention training had an advantage in the diversity between games, as compared to control (to avoid potential active effects in the latter). It is possible that this difference was (partially) responsible for the current transfer effects to general RT, rather than the specific content of the games. However, this observation does not negate the substantially greater demand for task switching within the flexibility games, nor the differential effects on RT and CRN measures as described above. Moreover, at least in terms of subjective experience (e.g., reported enjoyment and motivation) and training adherence, the data seemed to indicate no differences between the three trainings. Additionally, while analysis of the error-locked negativity (Ne/ERN; Band et al., 2009; Falkenstein, Hohnsbein, Hoormann, & Blanke, 1990) failed to show the expected increase after training, exploratory analysis did reveal an increase in the error-locked positivity (Pe; Falkenstein et al., 1994) specifically for flexibility as compared to both other trainings. This Pe effect might reflect an increased conscious awareness of errors (Nieuwenhuis et al., 2001), which can in turn promote the employment of different cognitive strategies. In sum, while both experimental trainings benefited general RT performance, ERP findings suggest different underlying mechanisms.

Third, some of the current null-effects for ERPs might have been due to practical issues with the collected data. For instance, in contrast to our prediction and the findings by Gajewski and Falkenstein (2012), we did not find training effects on error-related negativity Ne/ERN, likely due to the low percentage of errors. This difference might have partly been due to our young adult sample, as opposed to the elderly adult participants tested by Gajewski and Falkenstein. The presence of generally impaired cognitive resources in older adults compared to younger adults might also explain the non-replication of P3b amplitude effects.

Fourth, our findings might underestimate the potential benefits of training, for instance with regard to improvements in local switch costs, due to the at-home training setting. A meta-analysis by Lampit and colleagues (2014) found a generally reduced effectivity of at-home as compared to lab or group-based cognitive training interventions for older adults. At the same time, these potential limitations do emphasize the relevance of the current findings: online GCCT using easily accessible games can improve targeted and untargeted cognitive functions. Logical next-steps would include investigating both long-term effects as well as far transfer to real-world performance, such as academic performance or performance at work-related tasks.

## Conclusion

We have shown that targeted cognitive training using online games has the potential for both near-transfer and far-transfer effects to a measure of cognitive flexibility. Participants show generally faster responses, without a loss in accuracy, on the task-switch paradigm after receiving cognitive flexibility or attention training, as compared to an active control group. Training-induced changes in response times and in multiple ERP components suggested that training schedules had distinct effects on underlying neural mechanisms. Flexibility training in particular engendered more efficient conflict monitoring, while attention training seems most beneficial when low task difficulty undermines sustained attention. Surprisingly, switch-specific benefits were not found however. In sum, these results provide tentative encouragement for the use of at-home online brain-training games in targeted training schedules, and help shed light on some of the neurocognitive mechanisms underlying induced improvements. Finally, these findings suggest that an ideal GCCT would perhaps incorporate an even greater diversity of task-rules and goals both within and between games.

## Footnotes

1 Exploratory analysis of N2 and P3b peak latencies did not yield any significant effects including Session × Group (all *p* > 0.1).

2 Exploratory analysis with a 10 errors or more criterion (N_subjects_ = 65) and a 30-error criterion (N_subjects_ = 50) yielded qualitatively similar results.
